# Lateral Gene Transfer Dynamics in the Ancient Bacterial Genus *Streptomyces*

**DOI:** 10.1128/mBio.00644-17

**Published:** 2017-06-06

**Authors:** Bradon R. McDonald, Cameron R. Currie

**Affiliations:** a Department of Bacteriology, University of Wisconsin—Madison, Madison, Wisconsin, USA; b DOE Great Lakes Bioenergy Research Center, University of Wisconsin—Madison, Madison, Wisconsin, USA; Northern Arizona University

**Keywords:** antibiotics, evolutionary genomics, horizontal gene transfer, molecular clock, species concepts

## Abstract

Lateral gene transfer (LGT) profoundly shapes the evolution of bacterial lineages. LGT across disparate phylogenetic groups and genome content diversity between related organisms suggest a model of bacterial evolution that views LGT as rampant and promiscuous. It has even driven the argument that species concepts and tree-based phylogenetics cannot be applied to bacteria. Here, we show that acquisition and retention of genes through LGT are surprisingly rare in the ubiquitous and biomedically important bacterial genus *Streptomyces*. Using a molecular clock, we estimate that the *Streptomyces* bacteria are ~380 million years old, indicating that this bacterial genus is as ancient as land vertebrates. Calibrating LGT rate to this geologic time span, we find that on average only 10 genes per million years were acquired and subsequently maintained. Over that same time span, *Streptomyces* accumulated thousands of point mutations. By explicitly incorporating evolutionary timescale into our analyses, we provide a dramatically different view on the dynamics of LGT and its impact on bacterial evolution.

## IMPORTANCE

Tree-based phylogenetics and the use of species as units of diversity lie at the foundation of modern biology. In bacteria, these pillars of evolutionary theory have been called into question due to the observation of thousands of lateral gene transfer (LGT) events within and between lineages. Here, we show that acquisition and retention of genes through LGT are exceedingly rare in the bacterial genus *Streptomyces*, with merely one gene acquired in *Streptomyces* lineages every 100,000 years. These findings stand in contrast to the current assumption of rampant genetic exchange, which has become the dominant hypothesis used to explain bacterial diversity. Our results support a more nuanced understanding of genetic exchange, with LGT impacting evolution over short timescales but playing a significant role over long timescales. Deeper understanding of LGT provides new insight into the evolutionary history of life on Earth, as the vast majority of this history is microbial.

## INTRODUCTION

The bacterial domain encompasses prodigious diversity generated over billions of years of evolution (1). Despite the critical role that bacteria play in shaping nearly every aspect of life on Earth, understanding the complex evolutionary processes that generate this diversity remains a challenge. In contrast to sexual reproduction in eukaryotes, bacteria were originally thought to undergo strict clonal cell division with little to no genetic exchange. The discovery of conjugative plasmids (2), and later transformation and transduction (3), suggested that genetic exchange plays a role in bacterial evolution. The subsequent linking of nonhomologous lateral gene transfer (LGT) to important bacterial phenotypes, such as antibiotic resistance (4) and virulence (5), resulted in the recognition of LGT as a driving force in bacterial evolution. With the advent of comparative genomics and the identification of significant gene content differences between related bacteria (6), the prevailing view of rampant exchange of genes across bacteria emerged. Expanding on this view, some have argued that genetic exchange is so rampant that bacterial species do not exist as discrete entities (7) and their evolutionary histories fit a web of life model rather than a tree of life (8–11).

The disruptive impact of genetic exchange largely depends on several factors, including the degree to which barriers to LGT structure the exchanges between distantly related lineages. At larger phylogenetic scales, gene transfers have been shown to be more common within than between phyla, and some phyla exchange genes more frequently than others (12, 13). At the population level, both geographic distribution and sequence dissimilarity can lead to reduced rates of homologous recombination (14, 15). Analyses that span the species level to intermediate, genus-level diversity provide opportunities to investigate the combined effects of LGT and mutation across physiologically similar organisms that are diverging, either due to neutral processes or due to selective pressures in the different ecological niches that they occupy. This has the advantage of providing sufficient diversity to reliably detect LGT and identify trends that occur over evolutionary timescales, such as the loss of acquired genes that are selectively neutral or mildly deleterious (16–18). By including sufficient sampling of closely related organisms, it also reduces the amount of variation due to differences in core physiology between organisms in the data set. Focusing on a single genus that has broadly similar life history traits and is found in diverse environments enables easier detection of rapidly evolving diversity and ecologically relevant variation.

The ubiquitously distributed bacterial genus *Streptomyces* provides an excellent model for intermediate-scale analysis of genetic exchange and mutation. These diverse filamentous bacteria have been isolated from soil, marine, and host-associated environments, with ecological roles ranging from plant biomass degradation (19, 20) to defensive mutualisms with eukaryotes (21). Complex interactions between *Streptomyces* and other organisms are often characterized by the production of natural products (22), which have been mined for drug discovery for decades (23). Here, we utilize phylogenomic analyses combined with molecular clock dating to investigate the temporal scale of *Streptomyces* genomic and phylogenetic diversity, focusing on nonhomologous LGT and point mutations.

## RESULTS

**FIG 1:**
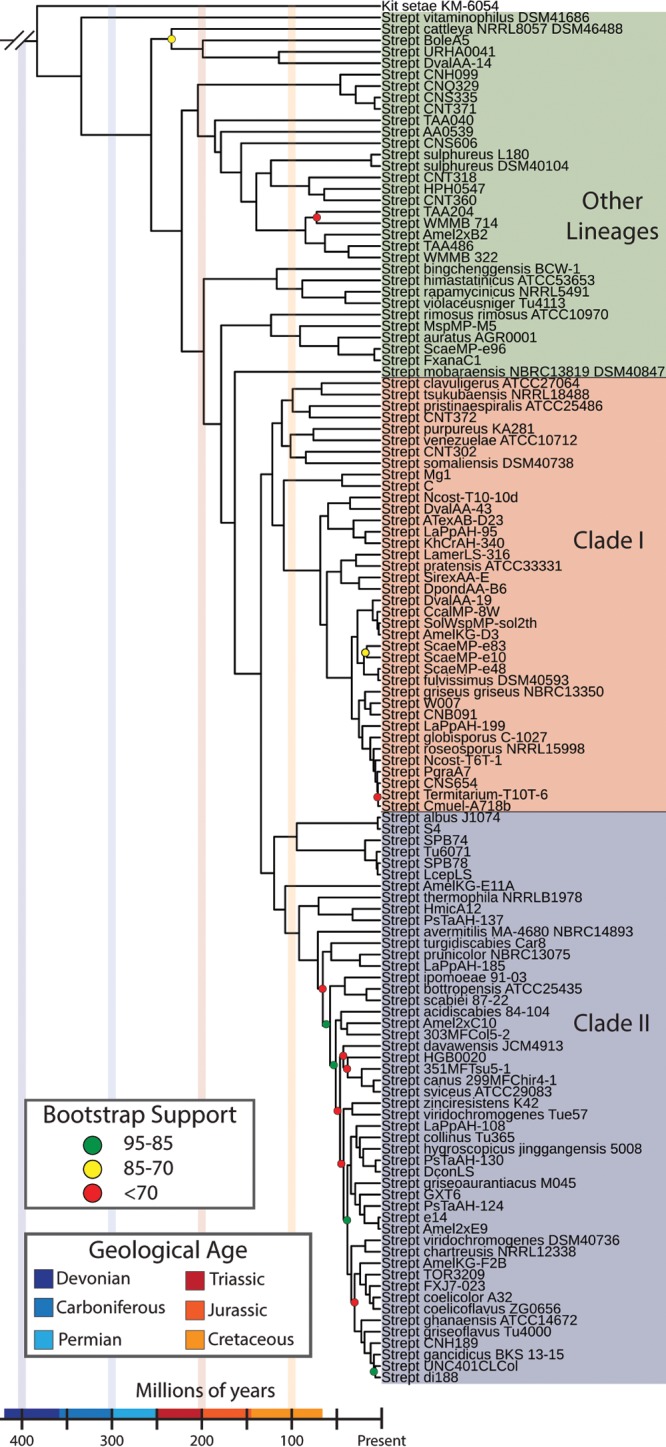
Genome-based phylogeny and molecular clock for the genus *Streptomyces*. TIGRFAM-based multilocus phylogeny of *Streptomyces* using 94 universally conserved housekeeping genes. Branch lengths indicate Reltime-estimated divergence times. Bootstrap values are shown by colored circles on all nodes with values of ≤95. *Streptomyces* and *Kitasatospora* are abbreviated as Strept and Kit, respectively.

**FIG 2:**
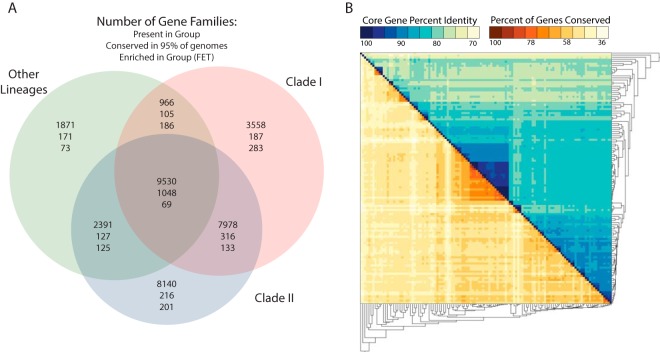
*Streptomyces* genome content conservation and divergence. (A) Proteinortho gene families present, conserved, and enriched in three groups of *Streptomyces* (clade I, clade II, and other lineages; based on Fig. 1). Gene family enrichment within subsets of *Streptomyces* was determined using Fisher’s exact test. (B) Pairwise comparison of conserved Proteinortho gene family percentage and TIGRFAM core gene sequence percent identity.

Based on our pan-genomic analyses of 122 *Streptomyces* genomes, 80 publicly available genomes and 42 additional genomes sequenced for this study, we identified significant phylogenetic and genomic diversity. The 42 strains chosen for genome sequencing were selected to obtain genome coverage across the genus; we selected strains to fill in gaps based on the phylogenetic location of the publicly available genomes in a 16S rRNA gene phylogeny (see Fig. S1 in the supplemental material). Using this expanded genomic data set, multilocus phylogenies were generated using both a traditional multilocus approach based on 94 housekeeping genes (Fig. 1, Fig. S2) and an alternative gene tree consensus-based approach implemented in ASTRAL-II (24) (Fig. S3). Both phylogenies are largely congruent, with two major monophyletic clades of *Streptomyces* containing 88 genomes (here referred to as clade I and clade II) and a number of other *Streptomyces* lineages containing the remaining 34 genomes (Fig. 1; also Fig. S2). These clades did not match the genome distribution on the 16S rRNA gene phylogeny, likely due to the poor resolution of 16S at finer phylogenetic scales. Most *Streptomyces* isolates from marine environments are found in more ancient lineages, suggesting a possible marine origin for the genus. Further, many strains in clade I were isolated from insect-associated niches (Fig. S2). Support values were high across both phylogenies with the exception of internal nodes in clade II, where tree topology differed in poorly supported nodes between the two phylogenetic methods. Exploring gene content, we identified a total of 39,893 gene families across the genus. Of these, 1,048 were conserved in 95% of *Streptomyces* genomes in the data set (Fig. 2). Each of the two major clades contained ~200 gene families that were conserved in the respective group but not the others. Each *Streptomyces* clade also contained a large number of unique genes, with 3,558 unique genes in clade I and 8,140 in clade II. Of these, a small percentage (5% in clade I and 2.5% in clade II) were conserved across the group. Shared genome content was correlated with phylogenetic distance, as more closely related genomes shared a higher proportion of genes (Fig. 2B). Overall, the results of our gene content analysis, without incorporating divergence times, are consistent with the prevailing view of bacterial evolution: high gene content diversity driven by frequent LGT.

10.1128/mBio.00644-17.1 FIG S1  16S rRNA gene phylogeny of the genus *Streptomyces*. Genome strains are noted by color coding to the corresponding phylogenetic group (red for clade I, blue for clade II, and green for all other lineages) in which they occur in the genome-based phylogeny (Fig. 1). Several strains/species are labeled for reference. *Streptomyces* is abbreviated as Strept. Download FIG S1, TIF file, 0.7 MB. Copyright © 2017 McDonald and Currie. 2017 McDonald and Currie This content is distributed under the terms of the Creative Commons Attribution 4.0 International license.

10.1128/mBio.00644-17.2 FIG S2  Full multilocus tree of *Streptomyces* and outgroup *Actinobacteria*. This phylogeny includes the full set of genomes used for AnGST analysis. *Streptomyces* strains are colored by isolation habitat. Scale bar indicates molecular clock divergence times estimated by Reltime. *Streptomyces* is abbreviated as *Strept*. Download FIG S2, TIF file, 1 MB. Copyright © 2017 McDonald and Currie. 2017 McDonald and Currie This content is distributed under the terms of the Creative Commons Attribution 4.0 International license.

10.1128/mBio.00644-17.3 FIG S3  Gene tree reconciliation-based *Streptomyces* phylogeny generated with ASTRAL-II. TIGRFAM gene families used for gene tree construction were the same as those used for the multilocus analysis. *Streptomyces* is abbreviated as Strept. Download FIG S3, TIF file, 2.3 MB. Copyright © 2017 McDonald and Currie. 2017 McDonald and Currie This content is distributed under the terms of the Creative Commons Attribution 4.0 International license.

Since mutation and LGT are dynamic processes that occur through time, identifying the rate of these events is critical for understanding their impact on the long-term evolution of a bacterial lineage. We estimated divergence times across the *Streptomyces* phylogeny using cyanobacterial fossils (25, 26), the estimated origin of life on Earth (27, 28), and the *Escherichia-Salmonella* divergence time (29) as calibration points for relaxed molecular clock analysis (Fig. S4; Table S1). Our analyses inferred that the genus *Streptomyces* diverged from *Kitasatospora* approximately 382 million years ago (mya) (confidence interval [CI], 250 to 514 mya), in the late Devonian period, and the two major clades diverged approximately 132 mya (CI, 87 to 177 mya), in the early to mid Cretaceous period. These divergence times also allow us to approximate the time span required for strains to diverge by 1% amino acid identity. Among 11 pairs of strains separated by 1% amino acid divergence in the core genes used for the phylogeny, the average divergence time is 8 ± 2.5 my.

10.1128/mBio.00644-17.4 FIG S4  Genome-based phylogeny and molecular clock for the domain *Bacteria*. Genome-based phylogeny of *Bacteria* used to calibrate *Streptomyces* divergence times. All unlabeled nodes have a bootstrap value of ≥95. Numbered nodes refer to Reltime divergence times that are compared to the molecular clock analysis performed in the work of Battistuzzi et al. (42), found in Table S1. *Streptomyces* is abbreviated as Strept. Download FIG S4, TIF file, 0.9 MB. Copyright © 2017 McDonald and Currie. 2017 McDonald and Currie This content is distributed under the terms of the Creative Commons Attribution 4.0 International license.

10.1128/mBio.00644-17.5 TABLE S1  Comparison of molecular clock estimated divergence times between Reltime (this study) and the work of Battistuzzi et al. (42) in millions of years. Node numbers refer to labels in Fig. S4. Download TABLE S1, DOCX file, 0.04 MB. Copyright © 2017 McDonald and Currie. 2017 McDonald and Currie This content is distributed under the terms of the Creative Commons Attribution 4.0 International license.

**FIG 3:**
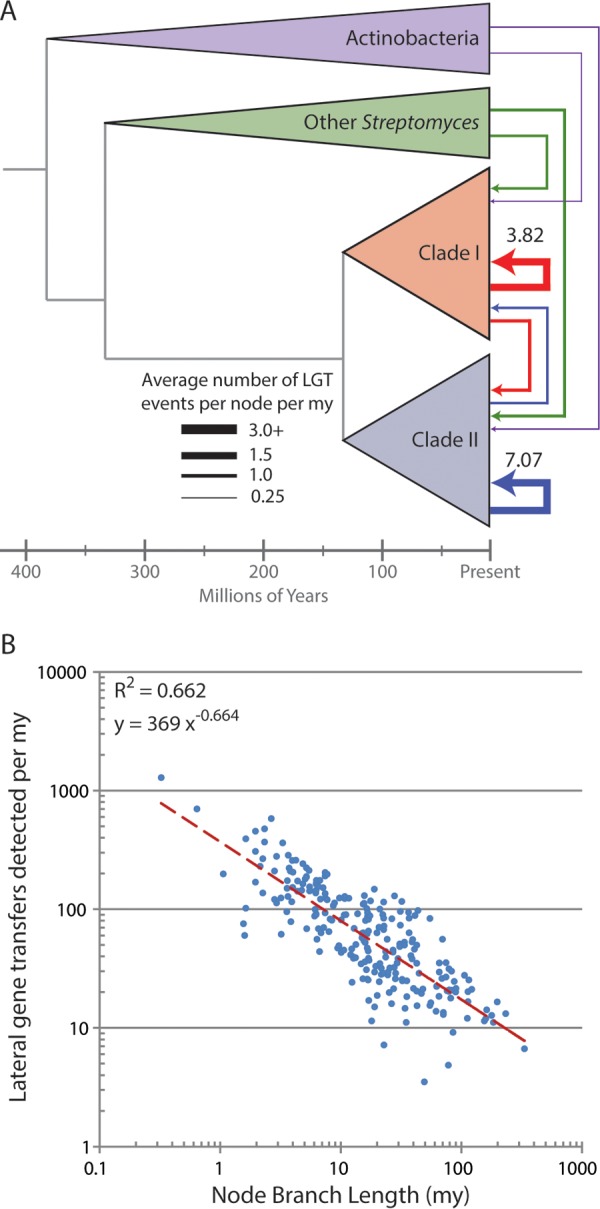
Rate of lateral gene transfer in *Streptomyces*. (A) Average rate of LGT across the *Streptomyces* phylogeny. Line thickness indicates the average number of detected LGT events per genome (including ancestral reconstructions) per million years from each source. Rates greater than 3 are labeled, and all rates appear in Table S2 in the supplemental material. (B) Detected rate of LGT on each branch of the phylogeny. Detected LGT rate is negatively correlated with branch length.

Investigation of LGT dynamics in *Streptomyces* revealed both functional and phylogenetic biases in gene transfer events. In total, we inferred 320,263 genes laterally acquired by *Streptomyces* lineages using the gene tree reconciliation approach implemented in AnGST (30). Gene functional classes overrepresented in LGT events consisted of secondary metabolism and xenobiotic metabolism (Table S2). Core biological functions such as transcription and translation were underrepresented (Table S3). Combined with the molecular clock dating, we estimate that overall rates of detectable LGT events per node into clade I or clade II are 5.93 and 9.08 per my, respectively (Fig. 3A; also Table S4). Nodes in clade I and clade II were significantly more likely to receive a gene transferred from a member of their own clade than from another source (*P* < 1e−5, Fisher’s exact test), with rates of 3.82 and 7.07 per million years for clades I and II, respectively. The estimated transfer rate per node from clade I to clade II is 1.08 transfers per my, and that from clade II to clade I is 1.43 per my. Inferred transfers of genes from other *Streptomyces* lineages to a genome in one of the major clades are significantly less common, occurring at approximately once every 2 million years. Acquisition of genes from different actinobacterial genera occurred even less frequently, at once every 3 million years for clade II and once every 5 million years for clade I.

10.1128/mBio.00644-17.6 TABLE S2  KEGG gene classes overrepresented in lateral gene transfer events within the bacterial genus *Streptomyces*. Download TABLE S2, DOCX file, 0.04 MB. Copyright © 2017 McDonald and Currie. 2017 McDonald and Currie This content is distributed under the terms of the Creative Commons Attribution 4.0 International license.

10.1128/mBio.00644-17.7 TABLE S3  KEGG gene classes underrepresented in lateral gene transfer events within the bacterial genus *Streptomyces*. Download TABLE S3, DOCX file, 0.05 MB. Copyright © 2017 McDonald and Currie. 2017 McDonald and Currie This content is distributed under the terms of the Creative Commons Attribution 4.0 International license.

10.1128/mBio.00644-17.8 TABLE S4  Rate of lateral gene transfer within and into clade I and clade II, as shown in Fig. 2. Download TABLE S4, DOCX file, 0.03 MB. Copyright © 2017 McDonald and Currie. 2017 McDonald and Currie This content is distributed under the terms of the Creative Commons Attribution 4.0 International license.

To investigate how the loss of neutral or deleterious genes acquired through LGT impacts estimates of gene transfer rates, we calculated transfer rate versus branch length across the *Streptomyces*. We found that the rate of detectable LGT events per million years is negatively correlated with branch length (Fig. 3B), and the rate can be approximated relative to branch length using a power law function (α = −0.664 and *R*^2^ = 0.662). KEGG (31, 32) genes with functions involved in replication and repair, translation, cell growth and death, and mobile elements make up a greater proportion of LGT events in short-branch-length nodes than in long-branch-length nodes (two-sided *t* test, *P* values of 0.009, 0.018, 0.019, and 0.027, respectively). This suggests that the lower number of detected LGT events involving core genes is due to stronger selection against transferred core genes, not a reduced number of actual transfers in these categories. It also suggests that mobile elements are gained and lost more rapidly on evolutionary timescales relative to other gene classes, which is consistent with expectations based on their biology.

**FIG 4:**
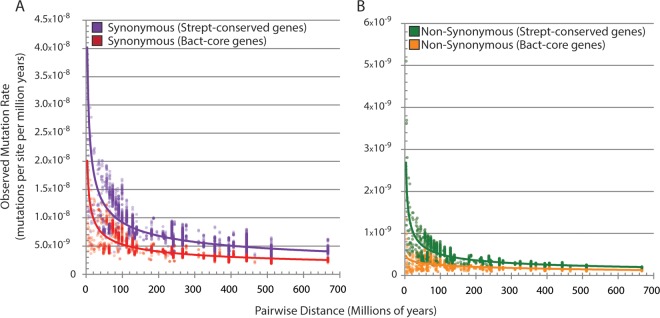
Rate of point mutations in TIGRFAM gene families. Bact-core genes consist of 94 housekeeping TIGRFAM gene families conserved across bacteria. *Strept-*conserved genes consist of 705 TIGRFAM gene families that are found in 95% of our *Streptomyces* genome data set. (A) The observed rate of synonymous point mutations varies by gene data set and pairwise distance. (B) The observed rate of nonsynonymous sites also varies by data set and pairwise distance, but to a lesser degree than that of synonymous sites.

We also investigated the relative contribution of point mutation versus LGT to *Streptomyces* diversity over time by calculating the rate of synonymous and nonsynonymous point mutations per million years in two different sets of conserved TIGRFAM (33) gene families: 705 families conserved across all *Streptomyces* (*Strept*-conserved) and the 94 universally conserved genes used to generate our phylogenies (Bact-core). Observed mutation rates differed between the two sets of genes that we analyzed, particularly for synonymous mutations (Fig. 4). The synonymous mutation rate was 1.5- to 2-fold higher in *Strept-*conserved genes than in the Bact-core genes. Similarly to the LGT rate, observed rates of both synonymous and nonsynonymous mutations are also influenced by the evolutionary distance between genome pairs; the synonymous mutation rate appears much higher in closely related genome pairs, while the difference in observed nonsynonymous mutation rates is less dramatic but still apparent. Using only pairwise comparisons of genomes separated by less than 100 my from the *Strept-*conserved data set, the estimated median rate of synonymous mutations is 1.62 × 10^−8^ per site per year and the median nonsynonymous mutation rate is 1.78 × 10^−9^ per site per year. Extrapolating the ratio of synonymous and nonsynonymous sites in the gene sequences that we analyzed to total coding sequence length, we estimate that a total of 13,714 synonymous and 10,429 nonsynonymous mutations accumulate in *Streptomyces* lineages per million years.

**FIG 5:**
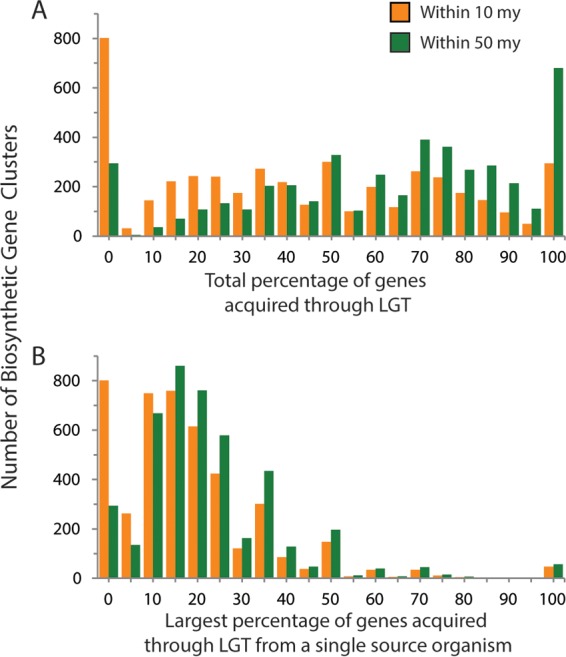
Sources of laterally transferred secondary metabolite biosynthesis genes. (A) Most biosynthetic gene clusters have acquired some genes through LGT within the last 50 my. (B) The vast majority of clusters appear to be a mix of genes from multiple sources, including being retained over millions of years within a lineage through vertical inheritance.

Given that natural product biosynthetic gene clusters (BGCs) were overrepresented in LGT events, and the known role that some of these play in producing small molecules that shape ecological interactions that are predicted to be under selection (34, 35), we examined the distribution and exchange of these secondary-metabolite-producing pathways. We identified a total of 4,945 natural product BGCs in *Streptomyces*; 1,759 clusters could be classified into 405 BGC families based on Pfam (36) domain content, while the domain structures of the other 3,186 were too dissimilar to match another *Streptomyces* cluster. We found that nearly all BGC families were nonrandomly distributed in *Streptomyces*, based on a branch-length permutation test: across the genus, 82.7% of BGC families were more phylogenetically restricted than expected by chance. This pattern also holds true at finer phylogenetic scales, as 79.2% and 76.1% of BGC families were more phylogenetically restricted than expected by chance within clade I and clade II, respectively. Interestingly, we also found very few cases of transfer and subsequent maintenance of complete BGC operons (Fig. 5). Analysis of BGC genes affected by LGT suggests that, at least over long evolutionary timescales, the vast majority of BGCs appear to have been affected by LGT. Specifically, our findings imply that 93% of BGCs acquired at least one gene through LGT within the last 50 my. However, only 57 BGCs had been acquired intact from one source, while the other BGCs were composed of a mixture of genes from multiple sources, including vertically inherited genes. Of the BGCs entirely acquired from a single source, most have been acquired within the last 10 my.

## DISCUSSION

Using *Streptomyces* divergence times rather than sequence similarity provides a new perspective on the rate of LGT and its potential impact on bacterial evolution over small and large evolutionary timescales. Although we inferred over 300,000 gene transfer events across the *Streptomyces*, a single successful gene transfer every hundred thousand years per lineage is sufficient to generate the observed number of events given the huge time span encompassed by *Streptomyces* evolution. Further, gene transfers from distantly related *Streptomyces* or other *Actinobacteria* are orders of magnitude less frequent than transfers from closely related lineages, suggesting that distantly related bacterial lineages are typically genetically isolated from each other for tens to hundreds of thousands of years at a time. We also find a strong effect of evolutionary distance between sampled genomes on the inferred rate of LGT. Our estimated transfer rate decreases steadily with branch length according to a two-thirds power law, likely due to acquisition and subsequent loss of genes with neutral or deleterious fitness effects (16). This suggests our calculated average LGT rate primarily measures acquisition rates for genes that are retained over evolutionary time. A similar correlation between evolutionary distance and inferred LGT rate was also found in *Pseudomonas syringae*, using percent amino acid divergence as the measure of distance rather than time (37). Overall, these results stand in contrast to the prevailing view that successful LGT events are rampant over short evolutionary time spans. The inferred rate of LGT accumulation may be impacted by taxon sampling, accurate species tree reconstruction, and LGT detection methods. Also, our analysis focused solely on the actinobacterial genus *Streptomyces*; further research is needed to determine how LGT dynamics vary over time in different lineages of bacteria. Nevertheless, given that our estimates of genomic diversity and total LGT events, using traditional pan-genome approaches, identify genome content patterns consistent with previous work across bacteria (e.g., ~300,000 LGT events), *Streptomyces* bacteria are unlikely to be outliers with respect to LGT rates.

We also show that approximately 23,000 point mutations accumulate per million years. Although each point mutation changes only a single base while an LGT event may involve the addition of thousands of base pairs, the phenotypic effect of any one mutation or LGT event is difficult to predict. A single point mutation in an active site may completely change an enzyme’s function, while an acquired gene that is not expressed may have no phenotypic effect. Our data suggest that while positively selected LGT events provide a rare but important source of genetic diversity, the vast majority of events that generate sequence diversity in *Streptomyces* are likely point mutations. Similar to the evolutionary distance effect on inferred LGT rate, pairwise comparisons between more distantly related organisms lead to lower estimated mutation rates, although this effect is weaker for point mutations than LGT events. As a result, the neutral rate of LGT is more difficult to infer from older lineages than the neutral point mutation rate. Estimated point mutation rates are also lower in genes that are conserved across most bacteria than in genes conserved in all *Streptomyces* species. The effects of evolutionary distance and gene data set on inferred mutation rate are stronger in synonymous sites, suggesting widespread fitness effects of synonymous mutations in *Streptomyces* core genes, as has been shown in a variety of bacteria and eukaryotes (38–41). These results suggest that comparisons of evolutionary event rates between bacterial groups can be strengthened through normalizing rates based on evolutionary distance between samples within each group.

Genes involved in the biosynthesis of natural products, the compounds that are critical for modern medicine and for which *Streptomyces* is well known, are among the genes most frequently acquired through LGT. In contrast to previous work in the related actinobacterial genus *Salinispora* (35), we found that most biosynthesis clusters were composed of genes apparently acquired from multiple sources rather than a single full-operon transfer event. These differences could be explained by the *Salinispora* data set being comprised of much more closely related genomes than our *Streptomyces* data set. Many BGC transfer events in *Streptomyces* might in fact be transfers of full clusters, followed by gene shuffling with other clusters in the same genome over evolutionary time. This would result in the scattered distribution of transferred genes that we observed in *Streptomyces* BGCs, without requiring different transfer dynamics than those seen in *Salinispora*.

Applying the temporal framework to *Streptomyces*, our results show that the biomedically and ecologically important genus *Streptomyces* is truly ancient; *Streptomyces* bacteria as a group are approximately as old as tetrapods and 60 my older than seed plants. The two major *Streptomyces* clades are approximately as old as flowering plants and older than the divergence of *Salmonella* and *Escherichia*. Therefore, despite being taxonomically defined as a genus, bacteria within lineages like the *Streptomyces* should not be considered “closely related.” Our analysis provides insight into intermediate-scale LGT dynamics, but our phylogenetic sampling does not enable us to investigate population-scale dynamics. Investigating processes that occur at this scale, including the short-term impacts of population size and drift on fixation and retention of genes acquired through LGT, will require intensive sampling of much more closely related bacterial strains, i.e., 99%-plus average nucleotide identity.

Our molecular clock analysis is consistent with several other molecular clock analyses performed across all bacteria (42) and *Actinomycetes* (43), using different methods and data sets. The majority of comparable nodes in our molecular clock analysis fall within the credibility intervals of previous rigorous analysis in bacteria performed by Battistuzzi et al. (42) (Table S1). Older nodes have larger confidence intervals and are more variable. This variability has limited effect on our analyses of *Streptomyces*, because only nodes that recently diverged, i.e., less than 400 my ago, were employed as reference points for the *Streptomyces* molecular clock that was used for downstream analyses. Although our molecular clock analysis is consistent with previous work, uncertainty in molecular clock dating generally means that the absolute rates of LGT that we identified are estimates. However, the extremely low rate of LGT means that our general conclusions remain valid even if the *Streptomyces* lineage is significantly younger than our analysis indicates. Further, the relative number of LGT events versus point mutations is robust to uncertainty in the molecular clock, since the two rates are calculated using the same divergence times.

Our results provide new insight into the paradox that, despite widespread LGT events, bacteria seemingly form “natural groups with coherent properties” (37, 44). The argument that LGT leads to significant divergence from the paradigm of vertical transmission of DNA from parent to daughter cell in bacteria emerged from a combination of high pan-genomic diversity and examples of gene acquisitions from distantly related organisms (45). However, we find strong evidence of phylogenetic biases in *Streptomyces* LGT, even in the most frequently transferred gene classes such as secondary metabolism. We also find evidence that many transferred genes may be selectively neutral or deleterious, leading to rapid turnover of acquired genes. An evolutionary model incorporating high neutral turnover of genes acquired through LGT is also supported by a positive correlation between genome fluidity and effective population size; bacterial lineages with higher synonymous nucleotide diversity also exhibit higher gene content diversity (46). These results suggest that LGT dynamics can be highly structured by phylogenetic (47) and physiological (48, 49) factors even within a genus, which limit its disruptive effect on bacterial evolution. Our incorporation of an absolute timescale reveals that the actual rate of successful transfer is many orders of magnitude lower than estimated bacterial generation times in nature (50, 51), and we show that thousands of point mutations may accumulate over that same timescale. Viewed from this perspective, the high absolute number of transfers detected in large bacterial genomic data sets may not be the result of high transfer rates but of long evolutionary time spans separating sampled strains. Because even bacteria in the same genus can be separated by tens to hundreds of millions of years, placing LGT in a temporal context also potentially explains the variable gene content patterns observed among closely related genomes without the need to invoke rampant LGT over short evolutionary time spans. Together, our results support a model of rare positively selected LGT events over millions of years driving gene content diversity in bacteria, with vertically inherited point mutations and homologous recombination dominating bacterial evolution over short evolutionary time spans and finer phylogenetic levels.

## MATERIALS AND METHODS

### Genome annotation.

In order to ensure consistent annotations across all genomes, protein-coding genes were predicted *de novo* using Prodigal (52). These were annotated using whole-protein HMMer3 (53) models generated from KEGG (31, 32) database gene families and TIGRFAM 13.0. They were also annotated using domain HMMer models from Pfam 27.0 and antiSMASH 2.0 (54). The TIGRFAM noise score cutoff and antiSMASH score cutoffs were used to remove false-positive hits, while KEGG hits were removed as false positives if their E value was greater than 1e–5, or if the difference in length between the HMMer model consensus and the protein was greater than 50%. rRNA genes were identified by performing BLAST v2.2.25 (55) analyses using sequences from the SILVA database (56). Proteins were also classified into families *de novo* based on sequence homology using Proteinortho v2 (57) with default parameters.

### Genome-based phylogenetics.

The *Streptomyces/Actinobacteria* multilocus phylogeny was generated using TIGRFAM annotated proteins. The 94 full TIGRFAM proteins in the “core bacterial protein” set (GenProp0799) were used as the molecular data set. The protein sequences with the top HMMer bitscore for each protein family in each genome were aligned using MAFFT (58). These protein alignments were then converted to codon alignments and were concatenated. Recombinant regions were identified using BratNEXTGEN (59) and masked to remove the potential confounding influence of homologous recombination on species tree topology. This removal did not have a significant influence on the final topology. RAxML-7.2.6 (60) was used to generate the phylogeny using the GTRgamma substitution model and 100 rapid bootstraps on the final, recombination-free alignment. The phylogeny of all bacteria used for molecular clock calibration was generated using a similar workflow, except that protein sequences were used to generate the phylogeny using the PROTGAMMABLOSUM62 substitution model in RAxML. Since genomes in this phylogeny were generally very distantly related to each other, no correction for homologous recombination was performed. The gene tree-based phylogeny was generated using ASTRAL-II. One hundred bootstrap alignments were generated for each of the core TIGRFAM families using RAxML. Phylogenies for each of these alignments were generated with FastTree 2.0 (61). These phylogenies were then used as the input data for ASTRAL-II.

### 16S rRNA gene phylogeny.

*Streptomyces* 16S rRNA gene sequences were obtained from RefSeq (62), along with 16S sequences extracted from the genomic data set and three cyanobacterial 16S sequences that were used as outgroups. These were aligned using MAFFT and then hand curated and trimmed to remove low-quality 16S sequences. The curated set of sequences was realigned and used to generate a phylogeny with FastTree.

### Molecular clock analyses.

Reltime (63) was used to approximate divergence times for two different phylogenies: the bacterial tree of life and the actinobacterial phylogeny containing the full set of *Streptomyces* genomes. The all-bacterium phylogeny and protein alignment described above were used as the input for Reltime. The algorithm was set to use “Many Clocks” and gamma-distributed rates with invariant sites. Approximate time intervals for the evolution of *Cyanobacteria* (2,500 to 3,500 million years ago) (25, 26), the divergence of *Salmonella* and *Escherichia* (50 to 150 million years ago) (29), and the origin of bacteria (3,500 to 3,800 million years ago) (27, 28) were used to calibrate the molecular clock found in Fig. S4 in the supplemental material. Using a single calibration point can correctly infer the divergence dates of the others with less than 20% error. The confidence intervals for the origins of *Actinobacteria* and *Streptomyces* and the divergence of *Streptomyces* clade I and clade II were then used to calibrate a second molecular clock analysis of the *Streptomyces*/*Actinobacteria* phylogeny found in Fig. 1 and S2.

### Lateral gene transfer analysis.

For each protein or domain database, the protein sequences for all gene families with more than 3 genes were aligned using MAFFT and then converted to codon alignments. Ten bootstrapped alignments were generated using RAxML for each gene family, and FastTree was used to generate a phylogeny for each bootstrapped alignment. These 10 bootstrap trees were then used as the gene tree inputs for each gene family in AnGST. Reconciliation event costs used were selected based on the criteria suggested in the original AnGST publication (30). Their weights for LGT and gene loss events were selected to minimize the mean change in genome size between parent and daughter nodes in the phylogeny. We analyzed LGT/loss weights of 10:1, 8:1, 5:1, and 3:1. Mean genome size changes for these weights were 4,344.12, 3,133.85, 1,159.95, and 702.71, respectively. Therefore, we used the 3:1 weights for the AnGST parameter settings. The *Streptomyces/Actinobacteria* molecular clock tree was provided as the species tree, and AnGST was run in ultrametric mode to avoid biologically improbable LGT events from extant genomes to deep ancestral nodes. The rate of LGT between or within subsets of *Streptomyces* was calculated as the number of genes acquired by genomes in the analyzed clade divided by the age of the last common ancestor of the clade in millions of years. LGT events affecting secondary metabolite clusters that occurred within a given time interval were identified by identifying genes that first appeared in a *Streptomyces* lineage within that interval. There is not a statistically significant difference in the inferred percentage of genes transferred into draft genomes versus complete genomes (*P* = 0.21, Mann-Whitney U), indicating that using predominantly draft genomes does not generate significant detection bias in our LGT analysis. The percentage of inferred gene losses is somewhat higher and more variable in draft genomes, a mean of 15.30% ± 8.91%, versus 10.41% ± 4.81% for complete genomes (*P* = 0.06, Mann-Whitney U). This is likely an artifact of variable-quality draft genome assemblies.

### Mutation rate estimation.

The concatenated codon alignment used to generate the *Streptomyces* phylogeny, along with concatenated codon alignments of all genes conserved in 95% of the *Streptomyces* genomes, was used to calculate point mutation rates. The number of synonymous and nonsynonymous mutations and sites was identified by the codeML package in PAML (64), and molecular clock divergence dates were used to calculate mutation rates per million years. We estimated the total number of synonymous and nonsynonymous sites across all coding regions by multiplying the number of sites in the TIGRFAM protein coding sequence by the average total length of protein coding sequence. Total number of mutations per million years was calculated based on the mutation rate and the estimated total number of sites per genome.

### Natural product biosynthesis cluster families.

Natural product biosynthetic gene clusters were predicted using the ClusterFinder algorithm and Pfam annotations (65). Genes that were found on the end of predicted clusters and had less than an 0.8 probability of being part of a cluster were removed. After this trimming, clusters with fewer than 3 genes and clusters lacking genes with an antiSMASH domain hit were removed. These curated clusters then were grouped into families using the modified Lin similarity metric (65, 66) with a Jaccard weight of 0.36, GK-gamma weight of 0.64, and overall similarity threshold of 0.7. We then processed the matches with the MCL algorithm, which was run with default parameters, to generate final cluster families. We used a subtree permutation approach to identify cluster families that were phylogenetically restricted, as in the work of Cafaro et al. (67). For each cluster family, we generated subtrees from the multilocus phylogeny containing only the genomes that possessed the cluster. We then compared the total branch length of this subtree to the branch-length distribution of 1,000 subtrees containing the same number of taxa, randomly sampled from the multilocus phylogeny. Cluster families were identified as phylogenetically restricted if their subtree total branch length was significantly less than the distribution mean by two-sided *t* test, with a *P* value cutoff of 1e–5.

### Accession number(s).

Genome sequence data are available from NCBI (http://www.ncbi.nlm.nih.gov). Accession numbers for all genomes are listed in Table S5. 

10.1128/mBio.00644-17.9 TABLE S5  NCBI accession numbers for all genomes used in this study. Download TABLE S5, DOCX file, 0.1 MB. Copyright © 2017 McDonald and Currie. 2017 McDonald and Currie This content is distributed under the terms of the Creative Commons Attribution 4.0 International license.
