# Supplementary figures and images for: Lateral Gene Transfer Dynamics in the Ancient Bacterial Genus Streptomyces
Source: mBio. 2017 Jun 6;8(3):e00644-17. doi: 10.1128/mBio.00644-17 (PMC5472806; doi:10.1128/mBio.00644-17)

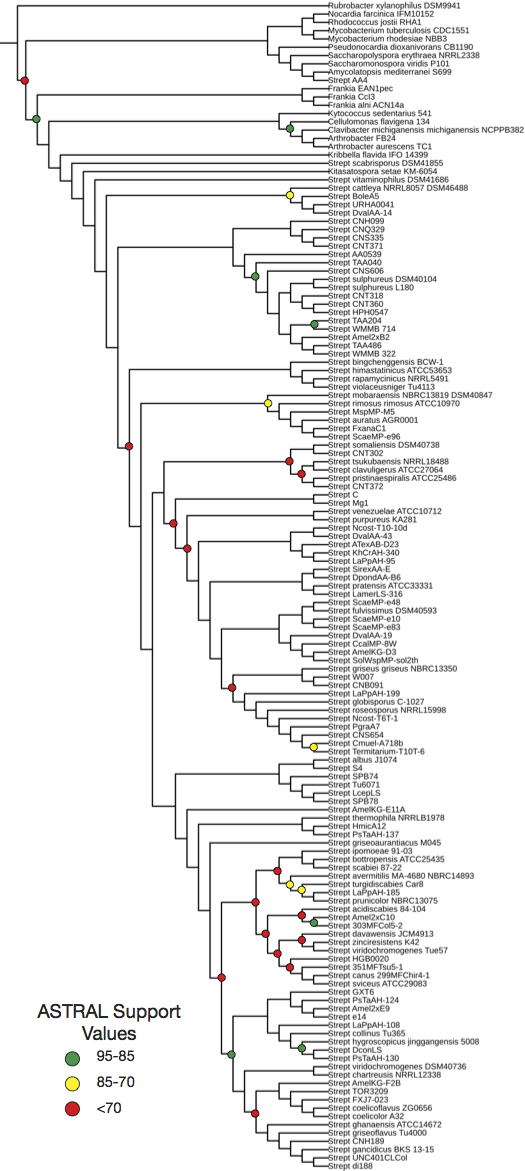

Supplement: FIG S3 [file mbo003173327sf3.tif]
