# Supplementary material for: Lateral Gene Transfer Dynamics in the Ancient Bacterial Genus Streptomyces
Source: mBio. 2017 Jun 6;8(3):e00644-17. doi: 10.1128/mBio.00644-17 (PMC5472806; doi:10.1128/mBio.00644-17)
Supplement: TABLE S1 [file mbo003173327st1.docx]

**Extended Data Table 1.**

| **Node** | **Reltime** | **BFU *et. al.*** | **Reltime Confidence Interval** | **BFU *et. al.* Credibility Interval** |
| --- | --- | --- | --- | --- |
| 1 | 3463 | 3054 | 1806-5121 | 2697-3490 |
| 2 | 3454 | 3051 | 1806-5121 | 2738-3434 |
| 3 | 3281 | 2784 | 1710-4852 | 2490-3203 |
| 4 | 2847 | 2508 | 1487-4208 | 2154-2928 |
| 5 | 2677 | 1924 | 1396-3958 | 1809-2525 |
| 6 | 2578 | 2743 | 1345-3811 | 2512-3076 |
| 7 | 87 | 102 | 45-130 | 57-176 |
