# Supplementary material for: Lateral Gene Transfer Dynamics in the Ancient Bacterial Genus Streptomyces
Source: mBio. 2017 Jun 6;8(3):e00644-17. doi: 10.1128/mBio.00644-17 (PMC5472806; doi:10.1128/mBio.00644-17)
Supplement: TABLE S2 [file mbo003173327st2.docx]

**Extended Data Table 2.**

| **KEGG category** | **P value** | **Odds ratio** |
| --- | --- | --- |
| Metabolism of Terpenoids and Polyketides | 4.70e-108 | 1.421 |
| Xenobiotics Biodegradation and Metabolism | 2.12e-127 | 1.351 |
| Biosynthesis of Other Secondary Metabolites | 1.50e-015 | 1.183 |
| Lipid Metabolism | 7.01e-017 | 1.120 |
