# Supplementary material for: Lateral Gene Transfer Dynamics in the Ancient Bacterial Genus Streptomyces
Source: mBio. 2017 Jun 6;8(3):e00644-17. doi: 10.1128/mBio.00644-17 (PMC5472806; doi:10.1128/mBio.00644-17)
Supplement: TABLE S3 [file mbo003173327st3.docx]

**Extended Data Table 3.**

| **KEGG category** | **P value** | **Odds ratio** |
| --- | --- | --- |
| Replication and Repair | 9.80e-95 | 0.638 |
| Transcription | 2.57e-20 | 0.652 |
| Nucleotide Metabolism | 4.61e-100 | 0.724 |
| Cell Growth and Death | 1.92e-13 | 0.762 |
| Glycan Biosynthesis and Metabolism | 5.43e-26 | 0.783 |
| Metabolism of Cofactors and Vitamins | 1.23e-50 | 0.839 |
| Energy Metabolism | 8.67e-51 | 0.842 |
| Translation | 6.41e-09 | 0.911 |
| Metabolism of Other Amino Acids | 3.96e-07 | 0.917 |
| Carbohydrate Metabolism | 1.22e-18 | 0.925 |
| Membrane Transport | 3.77e-07 | 0.943 |
| Signal Transduction | 0.038 | 0.973 |
| Amino Acid Metabolism | 0.012 | 0.981 |
