# Supplementary material for: Lateral Gene Transfer Dynamics in the Ancient Bacterial Genus Streptomyces
Source: mBio. 2017 Jun 6;8(3):e00644-17. doi: 10.1128/mBio.00644-17 (PMC5472806; doi:10.1128/mBio.00644-17)
Supplement: TABLE S4 [file mbo003173327st4.docx]

**Extended Data Table 4.**

|  | **Genes acquired per my** | |
| --- | --- | --- |
| **LGT Source** | **Clade I** | **Clade II** |
| Clade I *Streptomyces* | 3.816 | 1.082 |
| Clade II *Streptomyces* | 1.434 | 7.065 |
| Basal *Streptomyces* | 0.49 | 0.651 |
| Other Actinobacteria | 0.189 | 0.277 |
| Total | 5.929 | 9.075 |
