# Supplementary material for: Lateral Gene Transfer Dynamics in the Ancient Bacterial Genus Streptomyces
Source: mBio. 2017 Jun 6;8(3):e00644-17. doi: 10.1128/mBio.00644-17 (PMC5472806; doi:10.1128/mBio.00644-17)
Supplement: TABLE S5 [file mbo003173327st5.docx]

**Extended Data Table 5.**

| **Organism** | **Data source** | **NCBI Bioproject** |
| --- | --- | --- |
| *Streptomyces* sp AmelKG-F2B | Currie lab | in prep |
| *Streptomyces* sp. thermophila NRRLB1978 | Currie lab | in prep |
| *Streptomyces* sp. LaPpAH-185 | Currie lab | PRJNA302566 |
| *Streptomyces* sp. Ame12xE9 | Currie lab | PRJNA201126 |
| *Streptomyces* sp. Amel2xB2 | Currie lab | PRJNA195845 |
| *Streptomyces* sp. Amel2xC10 | Currie lab | PRJNA195842 |
| *Streptomyces* sp. AmelKG-D3 | Currie lab | PRJNA163015 |
| *Streptomyces* sp. AmelKG-E11A | Currie lab | PRJNA163025 |
| *Streptomyces* sp. ATexAB-D23 | Currie lab | PRJNA199252 |
| *Streptomyces* sp. BoleA5 | Currie lab | PRJNA169757 |
| *Streptomyces* sp. CcalMP-8W | Currie lab | PRJNA199236 |
| *Streptomyces* sp. Cmuel-A718b | Currie lab | PRJNA319231 |
| *Streptomyces* sp. DconLS | Currie lab | PRJNA319222 |
| *Streptomyces* sp. di188 | Currie lab | PRJNA319209 |
| *Streptomyces* sp. DpondAA-B6 | Currie lab | PRJNA195846 |
| *Streptomyces* sp. DvalAA-14 | Currie lab | PRJNA319210 |
| *Streptomyces* sp. DvalAA-19 | Currie lab | PRJNA319214 |
| *Streptomyces* sp. DvalAA-43 | Currie lab | PRJNA319215 |
| *Streptomyces* sp. e14 | Currie lab | PRJNA47353 |
| *Streptomyces* sp. e83 | Currie lab | PRJNA319216 |
| *Streptomyces* sp. FxanaC1 | Currie lab | PRJNA199074 |
| *Streptomyces* sp. KhCrAH-340 | Currie lab | PRJNA199243 |
| *Streptomyces* sp. LamerLS-316 | Currie lab | PRJNA169718 |
| *Streptomyces* sp. LaPpAH-108 | Currie lab | PRJNA199251 |
| *Streptomyces* sp. LaPpAH-199 | Currie lab | PRJNA302568 |
| *Streptomyces* sp. LaPpAH-95 | Currie lab | PRJNA199076 |
| *Streptomyces* sp. LcepLS | Currie lab | PRJNA319218 |
| *Streptomyces* sp. MspMP-M5 | Currie lab | PRJNA199249 |
| *Streptomyces* sp. Ncost-T10-10d | Currie lab | PRJNA319230 |
| *Streptomyces* sp. Ncost-T6T-1 | Currie lab | PRJNA163023 |
| *Streptomyces* sp. PgraA7 | Currie lab | PRJNA169756 |
| *Streptomyces* sp. PsTaAH-124 | Currie lab | PRJNA199254 |
| *Streptomyces* sp. PsTaAH-130 | Currie lab | PRJNA195843 |
| *Streptomyces* sp. PsTaAH-137 | Currie lab | PRJNA195844 |
| *Streptomyces* sp. ScaeMP-e10 | Currie lab | PRJNA199241 |
| *Streptomyces* sp. ScaeMP-e48 | Currie lab | PRJNA163017 |
| *Streptomyces* sp. ScaeMP-e96 | Currie lab | PRJNA163037 |
| *Streptomyces* sp. SirexAA-E | Currie lab | PRJNA72627 |
| *Streptomyces* sp. So1WspMP-so12th | Currie lab | PRJNA169755 |
| *Streptomyces* sp. SPB74 | Currie lab | PRJNA48415 |
| *Streptomyces* sp. SPB78 | Currie lab | PRJNA55819 |
| *Streptomyces* sp. Termitarium-T10T-6 | Currie lab | PRJNA319232 |
| *Streptomyces* sp. WMMB 322 | Currie lab | PRJNA187213 |
| *Streptomyces* sp. WMMB 714 | Currie lab | PRJNA187214 |
| *Streptomyces* albus J1074 | NCBI | PRJNA196849 |
| *Streptomyces* auratus AGR0001 | NCBI | PRJNA171646 |
| *Streptomyces* avermitilis MA4680 | NCBI | PRJNA57739 |
| *Streptomyces* bingchenggensis BCW-1 | NCBI | PRJNA82931 |
| *Streptomyces* coelicoflavus ZG0656 | NCBI | PRJNA180030 |
| *Streptomyces* coelicolor A3-2 | NCBI | PRJNA57801 |
| *Streptomyces* ghanaensis ATCC14672 | NCBI | PRJNA55543 |
| *Streptomyces* griseoaurantiacus M045 | NCBI | PRJNA66149 |
| *Streptomyces* griseoflavus Tu4000 | NCBI | PRJNA55831 |
| *Streptomyces* griseus NBRC13350 | NCBI | PRJNA58983 |
| *Streptomyces* hygroscopicus ATCC53653 | NCBI | PRJNA33605 |
| *Streptomyces* hygroscopicus jinggangensis 5008 | NCBI | PRJNA89409 |
| *Streptomyces* ipomoeae 91-03 | NCBI | PRJNA183480 |
| *Streptomyces* pristinaespiralis ATCC25486 | NCBI | PRJNA59511 |
| *Streptomyces* roseosporus NRRL15998 | NCBI | PRJNA55545 |
| *Streptomyces* scabiei 87-22 | NCBI | PRJNA46531 |
| *Streptomyces* scabrisporus DSM41855 | NCBI | PRJNA199206 |
| *Streptomyces* sp. 303MFCol5-2 | NCBI | PRJNA187949 |
| *Streptomyces* sp. 351MFTsu5-1 | NCBI | PRJNA187950 |
| *Streptomyces* sp. AA0539 | NCBI | PRJNA199548 |
| *Streptomyces* sp. AA4 | NCBI | PRJNA33599 |
| *Streptomyces* sp. acidiscabies 84-104 | NCBI | PRJNA77031 |
| *Streptomyces* sp. bottropensis ATCC25435 | NCBI | PRJNA176092 |
| *Streptomyces* sp. C | NCBI | PRJNA55823 |
| *Streptomyces* sp. canus 299MFChir4-1 | NCBI | PRJNA187948 |
| *Streptomyces* sp. cattleya NRRL8057 DSM46488 | NCBI | PRJNA78941 |
| *Streptomyces* sp. chartreusis NRRL12338 | NCBI | PRJNA72673 |
| *Streptomyces* sp. clavuligerus ATCC27064 | NCBI | PRJNA19249 |
| *Streptomyces* sp. CNB091 | NCBI | PRJNA199379 |
| *Streptomyces* sp. CNH099 | NCBI | PRJNA169791 |
| *Streptomyces* sp. CNH189 | NCBI | PRJNA169772 |
| *Streptomyces* sp. CNQ329 | NCBI | PRJNA190863 |
| *Streptomyces* sp. CNS335 | NCBI | PRJNA199338 |
| *Streptomyces* sp. CNS606 | NCBI | PRJNA195771 |
| *Streptomyces* sp. CNS654 | NCBI | PRJNA239507 |
| *Streptomyces* sp. CNT302 | NCBI | PRJNA199358 |
| *Streptomyces* sp. CNT318 | NCBI | PRJNA187951 |
| *Streptomyces* sp. CNT360 | NCBI | PRJNA187952 |
| *Streptomyces* sp. CNT371 | NCBI | PRJNA169776 |
| *Streptomyces* sp. CNT372 | NCBI | PRJNA199339 |
| *Streptomyces* sp. collinus Tu365 | NCBI | PRJNA171216 |
| *Streptomyces* sp. davawensis JCM4913 | NCBI | PRJEB184 |
| *Streptomyces* sp. fulvissimus DSM40593 | NCBI | PRJNA192408 |
| *Streptomyces* sp. FXJ7-023 | NCBI | PRJNA189794 |
| *Streptomyces* sp. gancidicus BKS 13-15 | NCBI | PRJNA186841 |
| *Streptomyces* sp. globisporus C-1027 | NCBI | PRJNA158251 |
| *Streptomyces* sp. GXT6 | NCBI | PRJNA178392 |
| *Streptomyces* sp. HGB0020 | NCBI | PRJNA72491 |
| *Streptomyces* sp. HmicA12 | NCBI | PRJNA169744 |
| *Streptomyces* sp. HPH0547 | NCBI | PRJNA169487 |
| *Streptomyces* sp. Mg1 | NCBI | PRJNA207881 |
| *Streptomyces* sp. mobaraensis NBRC13819 DSM40847 | NCBI | PRJNA188290 |
| *Streptomyces* sp. pratensis ATCC33331 | NCBI | PRJNA33771 |
| *Streptomyces* sp. prunicolor NBRC13075 | NCBI | PRJDB1071 |
| *Streptomyces* sp. purpureus KA281 | NCBI | PRJNA157921 |
| *Streptomyces* sp. rapamycinicus NRRL5491 | NCBI | PRJNA207502 |
| *Streptomyces* sp. rimosus rimosus ATCC10970 | NCBI | PRJNA182749 |
| *Streptomyces* sp. S4 | NCBI | PRJNA78151 |
| *Streptomyces* sp. somaliensis DSM40738 | NCBI | PRJNA81125 |
| *Streptomyces* sp. sulphureus DSM40104 | NCBI | PRJNA182442 |
| *Streptomyces* sp. TAA040 | NCBI | PRJNA187955 |
| *Streptomyces* sp. TAA204 | NCBI | PRJNA188326 |
| *Streptomyces* sp. TAA486 | NCBI | PRJNA190864 |
| *Streptomyces* sp. TOR3209 prodigal | NCBI | PRJNA198964 |
| *Streptomyces* sp. Tu6071 | NCBI | PRJNA66919 |
| *Streptomyces* sp. turgidiscabies Car8 | NCBI | PRJNA42361 |
| *Streptomyces* sp. UNC401CLCol | NCBI | PRJNA234927 |
| *Streptomyces* sp. URHA0041 | NCBI | PRJNA213783 |
| *Streptomyces* sp. viridochromogenes Tue57 | NCBI | PRJNA89157 |
| *Streptomyces* sp. vitaminophilus DSM41686 | NCBI | PRJNA199207 |
| *Streptomyces* sp. W007 | NCBI | PRJNA80699 |
| *Streptomyces* sulphureus L180 | NCBI | PRJNA200371 |
| *Streptomyces* sviceus ATCC29083 | NCBI | PRJNA59513 |
| *Streptomyces* tsukubaensis NRRL18488 | NCBI | PRJNA162933 |
| *Streptomyces* venezuelae ATCC10712 | NCBI | PRJNA177080 |
| *Streptomyces* violaceusniger Tu4113 | NCBI | PRJNA52609 |
| *Streptomyces* viridochromogenes DSM40736 | NCBI | PRJNA55829 |
| *Streptomyces* zinciresistens K42 | NCBI | PRJNA72955 |
| *Amycolatopsis mediterranei* S699 | NCBI | PRJNA170006 |
| *Aquifex aeolicus*VF5 | NCBI | PRJNA215 |
| *Arthrobacter aurescens* TC1 | NCBI | PRJNA12512 |
| *Arthrobacter* FB24 | NCBI | PRJNA12640 |
| *Bacillus subtilis subtilis* 168 | NCBI | PRJNA57675 |
| *Bacteroides vulgatus* ATCC 8482 | NCBI | PRJNA13378 |
| *Burkholderia cenocepacia* HI2424 | NCBI | PRJNA13918 |
| *Cellulamonas flavigena* 134 | NCBI | PRJNA19707 |
| *Clavibacter michiganensis* NCPPB382 | NCBI | PRJNA19643 |
| *Clostridium botulinum* A ATCC19397 | NCBI | PRJNA19517 |
| *Deinococcus radiodurans* R1 | NCBI | PRJNA57665 |
| *Enterococcus faecium* NRRLB-2354 | NCBI | PRJNA74725 |
| *Escherichia coli* K-12 subMG1655 | NCBI | PRJNA57779 |
| *Fibrobacter succinogenes* S85 | NCBI | PRJNA32617 |
| *Frankia alni* ACN14a | NCBI | PRJNA17403 |
| *Frankia* CcI3 | NCBI | PRJNA250957 |
| *Frankia* EAN1pec | NCBI | PRJNA13915 |
| *Gloeobacter violaceus* PCC 7421 | NCBI | PRJNA58011 |
| *Kitasatospora setae* KM-6054 | NCBI | PRJNA77027 |
| *Kribbella flavida* IFO 14399 | NCBI | PRJNA21089 |
| *Kytococcus sedentarius* 541 | NCBI | PRJNA21067 |
| *Lactobacillus fermentum* IFO 3956 | NCBI | PRJDA18979 |
| *Microcystis aruginosa* NIES-843 | NCBI | PRJDA27835 |
| *Mycobacterium rhodesiae* NBB3 | NCBI | PRJNA60027 |
| *Mycobacterium tuberculosis* CDC1551 | NCBI | PRJNA223 |
| *Nocardia farcinica* IFM10152 | NCBI | PRJNA13117 |
| *Nostoc punctiforme* PCC 73102 | NCBI | PRJNA216 |
| *Peptoclostridium difficile* CD196 | NCBI | PRJNA38037 |
| *Prevotella denticola* F0289 | NCBI | PRJNA49293 |
| *Prochlorococcus marinus* MIT 9312 | NCBI | PRJNA13910 |
| *Pseudonocardia dioxanivorans* CB1190 | NCBI | PRJNA40557 |
| *Rhodococcus jostii* RHA1 | NCBI | PRJNA13693 |
| *Rubrobacter xylanophilus* DSM9941 | NCBI | PRJNA10670 |
| *Saccharomonospora viridis* P101 | NCBI | PRJNA20835 |
| *Saccharopolyspora erythraea* NRRL2338 | NCBI | PRJEA18489 |
| *Salmonela enterica* *Typhimurium* LT2 | NCBI | PRJNA57799 |
| *Sinorhizobium fredii* NGR234 | NCBI | PRJNA59081 |
| *Streptococcus mutans* NN2025 | NCBI | PRJDA28997 |
| *Synechococcus elongatus* PCC 6301 | NCBI | PRJNA58235 |
| *Thermotoga maritima* MSB8 | NCBI | PRJNA57723 |
| *Vibrio cholerae* O1 El Tor N16961 | NCBI | PRJNA57623 |
